# Validation of a French language version of the Early Childhood Oral Health Impact Scale (ECOHIS)

**DOI:** 10.1186/1477-7525-6-9

**Published:** 2008-01-22

**Authors:** Shanshan Li, Jacques Veronneau, Paul J Allison

**Affiliations:** 1 Faculty of Dentistry, McGill University, Montreal, Canada

## Abstract

**Background:**

An English language oral health-related negative impact scale for 0–5 year old infants (the Early Childhood Oral Health Impact Scale [ECOHIS]) has recently been developed and validated. The overall aim of our study was to validate a French version of the ECOHIS. The objectives were to investigate the scale's: i) internal consistency; ii) test-retest reliability; iii) convergent validity; and iv) discriminant validity.

**Methods:**

Data were collected from two separate samples. Firstly, from 398 parents of children aged 12 months, recruited to a community-based intervention study, and secondly from 94 parents of 0–5 year-old children attending a hospital dental clinic. In a sub-sample of 101 of the community-based group, the scale was distributed a second time two weeks after initial evaluation. Internal consistency was evaluated through generation of Cronbach's alpha, test-retest reliability through intra-class-correlation coefficients (ICC), convergent validity through comparing scale total scores with a global evaluation of oral health and discriminant validity through investigation of differences in total scale scores between the community- and clinic-based samples.

**Results:**

Cronbach's alpha for both the child and family impact sections was 0.79, and for the whole scale was 0.82. The ICC was 0.95. Mean ECOHIS scores for parents rating their child's oral health as "relatively poor", "good" and "very good" were 10.8, 3.4 and 2.7 respectively. In the community- and clinic-based samples, the mean ECOHIS scores were 3.7 and 4.9 respectively.

**Conclusion:**

These results suggest this French language version of the ECOHIS is valid.

## Background

Children under five years of age can have many oral health problems, such as teething pains, early childhood caries (ECC) and dental trauma. Among these childhood oral health problems, ECC is common in many industrialized countries. However, the impact of oral ill-health on the functional, social and psychological well being of young children and their families has not been thoroughly investigated [1-3]. To do this, oral health-related quality of life (OHRQoL) instruments are required. In recent years OHRQoL instruments designed to investigate the impacts of oral health problems in children have begun to emerge [4-10], although until most recently, these instruments have been for 6–14 year old children. However, for children aged 0–5 years, an English language instrument to assess oral health-related negative impacts has recently been developed in the United States [11]. As with many such instruments, the Early Childhood Oral Health Impact Scale (ECOHIS) was developed in English and requires translation and validation in other languages if it is to be used in these alternative languages. We were interested in performing such work because we wanted to use the ECOHIS instrument to describe oral health problems in infants in Quebec, be able to make comparisons between oral health impacts in infants in Quebec and those elsewhere and also to potentially use the instrument as a tool to evaluate interventions. The goal of the study reported in this paper was to develop and validate a French language version of the ECOHIS so that it could be used among French-speaking populations. The specific objectives of the work reported in this paper were to translate the English version into French and then investigate the comprehensibility, internal consistency, test-retest reliability, convergent validity and discriminant validity of this French version of the ECOHIS.

## Methods

### The instrument

Details of the ECOHIS development and validation in its original English language version are reported elsewhere [11]. In summary, the instrument is reliable and able to discriminate between children with different levels of caries experience [11]. This ECOHIS consists of 13 questions and has two main parts: part one is the child impact section and part two is the family impact section. In the child impact section, there are four domains: child symptom, child function, child psychology, child self-image and social interaction. In the family impact section, there are two domains: parental distress and family function. The scale is scored using a simple Likert frequency type scale, with responses ranging from "Never" to "Very often" (equivalent to scores of 0–4) plus a "Don't know" option. Item scores are simply added to create a total scale score. This system creates a scale score range of 0–52, with higher scores indicating greater impacts and/or more problems.

### Translation into French

The ECOHIS was translated into French using the well-recognised forward-backward translation technique [12]. The process consisted of several stages. Firstly there was forward translation from English into French by two individuals whose first language is French, working independent of each other. Secondly, the two initial French versions were compared and revised through a consultation process involving the two translators and the principal investigator. Then the third French version produced by this process was back translated by two individuals whose first language is English, again working independently. Finally, the two back-translated English versions were compared with the original English version and final adjustments to the third French version made through consultation with all the translators involved, plus the principal investigator. This process resulted in a fourth French version of the ECOHIS, which was pilot tested in the target population so as to investigate the scale's comprehensibility, focusing on the wording of the items and the responses. This was a qualitative process among a convenience sample of 20 parents. This stage resulted in the revision of one item prior to the scale being ready for formal psychometric testing of its validity. A final point for the translation was that in our study, the referral time for the questions was the previous two weeks. This was different to the original instrument, which referred to the child's entire life. We chose a two week period because we were using the instrument in a prospective study with repeated, periodic evaluations, so a short term reference period rather than life time was more pertinent.

### The samples

Data used in the analyses reported in this paper came from two separate samples. Firstly, data were collected from 398 caregivers of children aged 12 months recruited to a community-based intervention study, and secondly from 94 parents of 0–5 year-old children attending a hospital clinic for dental treatment. In both samples, to be included, caregivers had to live with the child concerned 50% or more of the time and be comfortable reading and speaking French. This meant that they said "yes" when asked the question "Are you able to read and speak French?" and that they were able to read and sign a French language consent form. In the clinic-based sample, caregivers and their 0–5 year old children were approached while attending a children's hospital dental clinic for treatment of a "dental problem". "Dental problem" was defined through caregivers response to the question "Does your child have a dental problem that requires treatment?". The possible responses were "yes" or "no" and those responding "yes" were eligible for recruitment. Also, a sub-sample of 101 of the community-based group was mailed the French ECOHIS a second time two weeks after initial evaluation (with follow-up telephone calls for those who had not returned this second evaluation), to enable the evaluation of test-retest reliability. Socio-demographic data concerning the children and their caregivers were collected using self-complete questions routinely used in government surveys in Quebec. Data from these two samples are reported in Table 1. All parents recruited to this study signed a consent form. The study was submitted to and provided ethical approval by the Institutional Review Board of McGill University.

**Table 1:** Description of the community-based (mean age = 12 months) and clinic-based (mean age = 54 months) samples.

**Variable**	**Categories**	**Community-based sample (Total N = 398)**		**Clinic-based sample (Total N = 94)**	
		**N**	**%**	**N**	**%**

**Gender**	Male	187	46.9	58	61.7
	Female	211	53.1	36	38.3
**Level of education of child's mother**	Did not graduate high school	34	8.6	20	21.3
	Graduated high school	107	26.9	42	44.5
	College	143	36.0	26	27.7
	University	113	28.5	6	6.4
**Relationship of caregiver to child**	Biological mother	391	98.2	74	78.7
	Biological father	7	1.8	20	21.3
**Child's family yearly income**^a^	< $15,000	23	5.8	13	13.8
	$15,000 – $29,000	61	15.3	27	28.7
	$30,000 – $49,000	123	30.9	40	42.5
	>$49,000	191	47.9	14	14.9
**Last time mother saw dentist**	< 1 year ago	244	61.5	47	50.0
	1–2 years ago	94	23.8	26	27.7
	2–5 years ago	41	10.3	15	16.0
	> 5 years ago	18	4.5	6	6.4
**Treatment received at clinic**	Restoration	NA	NA	81	86.2
	Pulpectomy/pulpotomy			3	3.2
	Extraction			5	5.3
	Other			5	5.3

^a ^Child's family yearly income is measured in Canadian dollars

### Validation of the French version of the ECOHIS

#### Convergent validity

In order to examine convergent validity, an extra global oral health question ("Overall, how would you rate your child's oral health status?") was added at the end of ECOHIS. The possible responses to this question were "very good" "good" "fair" "poor" "very poor", and scores of 1–5 respectively were assigned to the aforementioned responses. Convergent validity was evaluated through investigating the correlation between ECOHIS total scores and the rating of the global oral health question, plus evaluating difference in mean ECOHIS scores by oral health status rating category. These analyses were performed with data from the community-based sample, using total ECOHIS score as a whole and child and parent impact sections separately. The underlying hypothesis was that parents who report high level of impacts in the scale should report poorer overall oral health than parents reporting low levels of impacts.

#### Discriminant validity

Our hypothesis was that the ECOHIS should be able to discriminate between children in the community with no immediate need for dental care and those in a dental clinic with an expressed need for dental care. Therefore, participants recruited from the community should have a lower ECOHIS score than participants with an expressed dental problem recruited at a dental clinic. The analysis of the different scores was performed using multiple linear regression analysis so as to control for age because, while subjects in the community sample were all approximately 12 months of age, those in the clinic-based sample varied in age between 6–60 months. These analyses were performed using total ECOHIS score as a whole and child and parent impact sections separately.

#### Internal consistency

Internal consistency was evaluated using data gathered from the community-based sample. It was estimated through generation of Cronbach's alpha for the child and family impact sections of the scale separately, plus the instrument as a whole. Item-scale and child-family scale correlations were evaluated through generation of Pearson correlation coefficients.

#### Test-retest reliability

This was evaluated using data gathered from the community-based sample. Two weeks after initial administration of the scale to the 398 participants, a subgroup of 101 participants was chosen at random (every 3^rd ^dyad in our list of participants was asked be part of this subgroup) to complete the scale a second time. In addition to the French ECOHIS, this subgroup of 101 caregivers was asked if the oral health of their child had changed during the previous two weeks. This was done using a self-complete question in the questionnaire that included the ECOHIS instrument, which was mailed to subjects. Only data from caregivers that reported no change in their child's oral health status were used to examine test-retest reliability. The intra-class correlation coefficient calculated using the INTRACC macro in SAS was used to evaluate test-retest reliability.

All data analyses were performed using the SAS program (SAS 7.0).

## Results

Table 1 shows the results of descriptive analyses of the sociodemographic information for the community-based and clinic-based samples. The mean age of the 94 subjects in the clinic-based sample was 54.3 months, with a range of 6–60 months. Tables 2 and 3 show the distribution of responses to the ECOHIS in the two samples. Eleven percent of subjects in the community-based sample and 15% of subjects in the clinic-based sample had at least one question with no response. Table 3 shows that in the community-based sample, items related to "pain" (46.3%), "sleeping" (25.3%) and "frustration" (36.8%) were reported most frequently in the child impact section of the scale. However, the distribution of responses to each question was skewed because most participants responded "never". Table 4 shows the distribution of responses in the clinic-based sample and permits the observation that, compared with the community-based sample, participants from clinic-based sample reported more oral health related problems, with more participants answering "very often". In the clinic-based sample, in the child impact section of the scale, the items related to problems with "pain" and "eating" were reported most frequently. In the family impact section, the level of impacts was higher with "feeling upset...." being reported most frequently. Once again, however, in the clinic-based sample the responses to each item were skewed towards subjects reporting "never" experiencing the problem.

**Table 2:** Distribution of French ECOHIS responses in the community-based sample (N = 398)

**Impacts**	**Never N (%)**	**Hardly ever N (%)**	**Occasionally N (%)**	**Often N (%)**	**Very often N (%)**	**Don't know N (%)**
Pain	187 (49.1)	18 (4.7)	92 (24.2)	59 (15.5)	25 (6.6)	17 (4.3)
Drinking	366 (94.1)	15 (3.9)	6 (1.5)	1 (0.3)	1 (0.3)	9 (2.3)
Eating	333 (84.3)	21 (5.3)	32 (8.1)	6 (1.5)	3 (0.8)	3 (0.8)
Pronouncing	372 (96.6)	9 (2.3)	2 (0.5)	0	2 (0.5)	13 (3.3)
Absence	382 (98.5)	3 (0.8)	3 (0.8)	0	0	10 (2.5)
Sleeping	260 (66.5)	32 (8.2)	72 (18.4)	16 (4.1)	11 (2.8)	7 (1.8)
Frustrated	201 (51.4)	46 (11.8)	106 (27.1)	24 (6.1)	14 (3.6)	7 (1.8)
Smiling	367 (93.6)	17 (4.3)	8 (2.0)	0	0	6 (1.5)
Talking	384 (98.0)	6 (1.5)	2 (0.5)	0	0	6 (1.5)
Upset	340 (86.7)	22 (5.6)	25 (6.4)	4 (1.0)	1 (0.3)	6 (1.5)
Guilty	378 (95.9)	10 (2.5)	6 (1.5)	0	0	4 (1.0)
Work	382 (97.5)	4 (1.0)	6 (1.5)	0	0	6 (1.5)
Financial	391 (99.2)	2 (0.5)	0	1 (0.3)	0	4 (1.0)

**Table 3:** Distribution of French ECOHIS responses in the clinic-based sample (N = 94)

**Impacts**	**Never N (%)**	**Hardly ever N (%)**	**Occasionally N (%)**	**Often N (%)**	**Very often N (%)**	**Don't know N (%)**
Pain	56 (62.9)	11 (12.4)	15 (16.9)	2 (2.1)	5 (5.3)	5 (5.3)
Drinking	72 (77.4)	9 (9.7)	7 (7.5)	1 (1.1)	4 (4.3)	1 (1.1)
Eating	69 (75.0)	5 (5.4)	13 (14.1)	2 (2.1)	3 (3.2)	2 (2.1)
Pronouncing	78 (85.7)	8 (8.5)	1 (1.1)	3 (3.2)	1 (1.1)	3 (3.2)
Absence	79 (85.9)	8 (8.7)	5 (5.4)	0	0	2 (2.1)
Sleeping	77 (82.8)	7 (7.5)	6 (6.5)	2 (2.1)	1 (1.1)	1 (1.1)
Frustrated	68 (73.1)	9 (9.7)	11 (11.8)	3 (3.2)	2 (2.1)	1 (1.1)
Smiling	80 (87.0)	5 (5.4)	5 (5.4)	2 (2.1)	0	2 (2.1)
Talking	81 (90.0)	6 (6.7)	1 (1.1)	2 (2.1)	0	4 (4.3)
Upset	63 (67.0)	10 (10.6)	14 (14.9)	3 (3.2)	4 (4.3)	0
Guilty	68 (73.9)	6 (6.5)	7 (7.61)	6 (6.5)	5 (5.4)	2 (2.1)
Work	76 (81.7)	5 (5.4)	6 (6.5)	3 (3.2)	3 (3.2)	1 (1.1)
Financial	79 (86.8)	8 (8.8)	4 (4.4)	0	0	3 (3.2)

With respect to the analysis of convergent validity, performed using data from the community-based sample, responses to the global oral health question were skewed strongly towards the "very good" response (very poor 0.3%; poor 0%; fair 2.1%; good 23.4%; very good 74.2%). As a result, we created three categories of response to the global oral health rating: those caregivers reporting their child's oral health as being "very poor", "poor" and "fair" versus those reporting it to be "good" and those reporting it to be "very good". The mean total French ECOHIS scores for these subjects in the "poor-to-fair", "good" and "very good" global oral health categories were 10.8, 3.4 and 2.7 respectively. In addition we investigated the Spearman correlation coefficient for the global rating and total ECOHIS score and found it to be a weak but significant correlation (r = -0.20; p = 0.004). The correlations for the global ratings with the child and parental impact sections of the ECOHIS were r = -0.15 (p = 0.013) and r = -0.18 (p = 0.008) respectively.

Table 4 shows the mean French ECOHIS scores for the total scale and different domains in the community- and clinic-based samples. In all cases the mean scores of the clinic-based sample were higher than those of the community-based sample. Multi-linear regression analysis of the correlates of the total ECOHIS score was performed and demonstrated that controlling for age and gender, the source of the sample (clinic- versus community-based) was strongly (parameter estimate = 3.61; r^2 ^= 0.12) and significantly (p < 0.0001) associated with the total ECOHIS score, with the clinic-based sample having a higher impact. This analysis also demonstrated that age was significantly associated with ECOHIS score (parameter estimate = 0.08; r^2 ^= 0.07; p < 0.0001), with impact increasing by age.

**Table 4:** Comparison of French ECOHIS scores for different domains in community-based and clinic-based samples

***Impacts***	***No of items***	***Range***	***Community Sample Mean ± Sd***	***Clinic Sample Mean ± Sd***
Child Symptoms	1	0–4	1.2 ± 1.4	1.7 ± 1.2
Child Function	4	0–16	0.5 ± 0.2	1.3 ± 1.2
Child Psychology	2	0–8	1.6 ± 1.0	1.8 ± 1.6
Self image and social interaction	2	0–8	0.1 ± 0.5	0.3 ± 0.1
**Child impact subscale**	**9**	**0–36**	**3.3 ± 1.7**	**4.9 ± 3.0**
Parental Distress	2	0–8	0.3 ± 0.8	1.3 ± 1.1
Family Function	2	0–8	0.1 ± 0.4	0.5 ± 0.2
**Parental impact subscale**	**4**	**0–16**	**0.4 ± 0.5**	**1.6 ± 0.8**

**Whole scale**	**13**	**0–52**	**3.7 ± 1.5**	**5.9 ± 3.1**

In examining the internal consistency of the French ECOHIS, using data from the community-based sample, we found Cronbach's alpha values of 0.79 and 0.79 for the child impact and family impact sections respectively, and 0.82 for the instrument as a whole. The Pearson correlation coefficient for the correlation of the child and family section scores was r = 0.54 (p < 0.0001). The item scale correlations were ranged between r = 0.21 – 0.73, were positive in nature and all were statistically significant.

Finally, the test-retest reliability of the French ECOHIS was examined through a sub-sample of the community-based sample completing the scale a second time two weeks following the first completion. There were 49/101 (48%) participants who reported no change in health status and returned the instrument with complete responses. Among these 49 subjects, intra-class correlation coefficients were 0.95 for the whole scale, 0.93 for the child impact section and 0.81 for the family impact section.

## Discussion

The aim of this study was to validate a French language version of the ECOHIS by examining its internal consistency, test-retest reliability, convergent validity and discriminant validity. The results of this validation process indicated that Cronbach's alpha was 0.79 for each of the child and family impact sections and 0.82 for the whole scale, the intra-class correlation coefficient was 0.95, total ECOHIS scores correlated with a global evaluation of oral health and the French ECOHIS was able to discriminate between children in the community with no expressed need for dental care and those in a dental clinic with an expressed need for dental care. Overall, therefore, in all the tests of validity to which we have subjected this French version of the ECOHIS, it has performed well. This indicates that it is a valid instrument when used by French-speaking caregivers of 0–5 year old children to describe the oral health impacts experienced their children and when used to discriminate between groups whose levels of problems are expected to be different.

Having made this inference, however, it is important to recognise the limitations of the work performed in terms of the methodology and analytic strategies used, the performance of the French ECOHIS and the extent of the validation tests. In terms of the methodological and analytical approaches, there are two limitations worth discussing. Firstly, the two study samples were convenience in nature and so cannot be said to represent any particular population. However, this is of secondary importance in tests of the validation of an instrument, where sampling should be more purposive and related to the needs of each element of the validation process [13]. The second methodological and analytical limitation worth mentioning concerns the means of testing the discriminant validity of the instrument. When testing the discriminant validity of the original version, the ECOHIS designers investigated the association between total scores and dmft in their sample [11]. We did not evaluate clinical indicators in our samples because the community-based sample was only 12 months old and so was likely to have extremely low levels of caries experience. Thus we conceived of an alternative hypothesis to test discriminative validity in the French version. The very different age ranges of the two samples we used was not ideal, however, we were able to control for this variable in our analysis by using multiple regression analysis. Age was indeed a highly significant predictor of the French ECOHIS score along with sample source (community- versus clinic-based). It is also interesting to note that yet another series of comparisons was used to test the discriminant validity of the CPQ8-10 and CPQ11-14: a comparison between children attending paediatric, orthodontic and craniofacial treatment clinics [4,5,7,8]. Beyond these methodological and analytical limitations, the techniques and strategies we used were standard.

In terms of the performance of the French ECOHIS, there are a number of limitations to be noted. The most important of these is the floor effect. The data were strongly skewed towards the no impact end of the scale, with 49–99% of subjects (depending upon the item) reporting "never" experiencing problems in the community-based sample and 63–90% of subjects reporting the same in the clinic-based sample. This is probably indicative of the subjects having genuinely low levels of problems but may be due to the instrument not being sensitive to problems that are experienced. In this respect, it is important to note that the results obtained using the French ECOHIS are similar to those obtained using the original version, which also had a strong floor effect [11]. Neither the original nor the French version had any ceiling effects. A second performance indicator for the French ECOHIS that is worth noting is the level of non-responses. As with the original version, we kept an "I don't know" response option, which is important, particularly during the validation phase of instrument development and use, so as to have an indication of the pertinence and comprehensibility of the items and to be sure that subjects have actively attempted to respond to the question rather than simply erroneously (or purposely) missed it. Scales or items with too many "I don't know" responses clearly have relevance or comprehensibility problems, while those with too many missing data have problems with the former and/or with the design/set-up of the scale. With respect to the French ECOHIS, 11% of participants in the community-based sample and 19% of subjects in the clinic-based sample respectively answered "I don't know" to one or more of the questions. No subject answered "I don't know" to all questions. The 11% figure for the community-based sample is a little higher than the 7% reported for the original ECOHIS [11] but the 19% figure for the clinic-based sample is much higher and may indicate that the relevance of the instrument in a clinic setting in parents with children with expressed dental needs and problems may be lower than in a community-based sample. Finally, with respect to the performance of the French ECOHIS, it is worth noting the extremely low levels of problems for the financial impact item in the samples used in this project. The subjects were recruited in Quebec, Canada, where routine dental examinations and treatments for children under 10 years old is paid for by the government, so this item may be of limited relevance.

Finally, with respect to the limitations in the extent of the validation tests, it is important to note that we have demonstrated that this French version of the ECOHIS possesses good internal consistency and external reliability, which are standard properties for any instrument, and it performs as expected with respect to convergent and discriminant validity. However, we have not tested its ability to evaluate treatments/interventions or predict future events. The instrument was not designed to perform these tasks but it is important to recognise its purposes and its limitations, although it may in the future be tested as an evaluative or other type of instrument in addition to its current descriptive and discriminative role.

Having acknowledged these limitations, it is also interesting to compare our findings with those of the validation of the original version of the ECOHIS. In the original US study, a convenience sample of 295 parents of 5-year-old children was chosen from five high income and three low income counties in North Carolina. Thus there was a clear difference in age between our community-based sample and the US sample. However, the three out of four of the most common impacts in both groups were the same. In our sample the most common impacts in the community-based sample were "pain", "frustration", "sleeping" and "eating", while in the US group they were "pain", "frustration", "eating" and "missed preschool" [11]. In the family impact section, the "upset" and "guilty" items were reported most frequently in the US group, while in our community-based sample all family impacts were negligible. However, in our clinic-based group, whose age was much more similar to that of the US sample, the family impact levels were comparable. As for the psychometric properties of the English and French versions, both were very good.

## Conclusion

In conclusion, the findings of our work suggest that this French language version of ECOHIS has good internal consistency test-retest reliability, convergent validity and discriminant validity. It is therefore appropriate to use it to describe OHRQoL in 0–5 year olds with French-speaking parents in Quebec and potentially in other French-speaking populations in the world. Using this French ECOHIS will also enable comparisons with English-speaking groups. However, this French ECOHIS has not been validated as an evaluative or predictive instrument so care would need to be taken if attempting to use it in these sorts of contexts.

## Abbreviations

ECOHIS: Early childhood oral health impact scale; ICC: Intra-class correlation coefficient; ECC: Early childhood caries; OHRQoL: Oral health-related quality of life

## Competing interests

The author(s) declare that they have no competing interests.

## Authors' contributions

SL performed the data analyses and wrote the first draft of the manuscript. JV contributed to the design of the study, recruited study sites for the project, recruited subjects for the study and contributed to the writing of the manuscript. PJA wrote the protocol for the study, oversaw data collection and data analyses and contributed to and supervised the writing of the submitted manuscript. All authors read and approved the final manuscript.
